# Preparation and characterization of a new graphite superconductor: Ca_0.5_Sr_0.5_C_6_

**DOI:** 10.1038/s41598-017-07763-y

**Published:** 2017-08-07

**Authors:** Saki Nishiyama, Hidenori Fujita, Masatoshi Hoshi, Xiao Miao, Takahiro Terao, Xiaofan Yang, Takafumi Miyazaki, Hidenori Goto, Tomoko Kagayama, Katsuya Shimizu, Hitoshi Yamaoka, Hirofumi Ishii, Yen-Fa Liao, Yoshihiro Kubozono

**Affiliations:** 1 Research Institute for Interdisciplinary Science, Okayama University, Okayama, 700-8530 Japan; 2 Center for Science and Technology under Extreme Conditions, Graduate School of Engineering Science, Osaka University, Osaka, 560-8531 Japan; 3 Research Laboratory for Surface Science, Okayama University, Okayama, 700-8530 Japan; 4 RIKEN SPring-8 Center, RIKEN, 1-1-1 Kouto, Sayo, Hyogo, 679-5148 Japan; 5 National Synchrotron Radiation Research Center, Hsinchu, 30076 Taiwan

## Abstract

We have produced a superconducting binary-elements intercalated graphite, Ca_x_Sr_1−x_C_y_, with the intercalation of Sr and Ca in highly-oriented pyrolytic graphite; the superconducting transition temperature, *T*_c_, was ~3 K. The superconducting Ca_x_Sr_1−x_C_y_ sample was fabricated with the nominal x value of 0.8, *i.e*., Ca_0.8_Sr_0.2_C_y_. Energy dispersive X-ray (EDX) spectroscopy provided the stoichiometry of Ca_0.5(2)_Sr_0.5(2)_C_y_ for this sample, and the X-ray powder diffraction (XRD) pattern showed that Ca_0.5(2)_Sr_0.5(2)_C_y_ took the SrC_6_-type hexagonal-structure rather than CaC_6_-type rhombohedral-structure. Consequently, the chemical formula of Ca_x_Sr_1−x_C_y_ sample could be expressed as ‘Ca_0.5(2)_Sr_0.5(2)_C_6_’. The XRD pattern of Ca_0.5(2)_Sr_0.5(2)_C_6_ was measured at 0–31 GPa, showing that the lattice shrank monotonically with increasing pressure up to 8.6 GPa, with the structural phase transition occurring above 8.6 GPa. The pressure dependence of *T*_c_ was determined from the DC magnetic susceptibility and resistance up to 15 GPa, which exhibited a positive pressure dependence of *T*_c_ up to 8.3 GPa, as in YbC_6_, SrC_6_, KC_8_, CaC_6_ and Ca_0.6_K_0.4_C_8_. The further application of pressure caused the rapid decrease of *T*_c_. In this study, the fabrication and superconducting properties of new binary-elements intercalated graphite, Ca_x_Sr_1−x_C_y_, are fully investigated, and suitable combinations of elements are suggested for binary-elements intercalated graphite.

## Introduction

Some graphite intercalation compounds show superconductivity, and have attracted serious attention because of their high superconducting transition temperatures (*T*_c_’s). The highest-onset superconducting transition temperature, *T*_c_^onset^, is currently 11.5 K at ambient pressure (0 GPa)^1, 2^ and 15.1 K at 7.5 GPa for CaC_6_^3^. However, despite much effort to make new graphite superconductors, no graphite superconductors with higher *T*_c_^onset^ values than 11.5 K have been synthesized. In fact, the *T*_c_ values of graphite superconductors prepared by the intercalation of alkali metal atoms thus far were 136 mK for KC_8_^4, 5^ and 23 mK for RbC_8_^4^. The graphite superconductors prepared by alkali earth or lanthanide atoms were SrC_6_ (*T*_c_ = 1.65 K)^6^, BaC_6_ (*T*_c_ = 65 mK)^7^ and YbC_6_ (*T*_c_ = 6.5 K)^1^. Furthermore, binary-elements intercalated graphite was first achieved in KHgC_8_ (*T*_c_ = 1.9 K)^8^. Subsequently, some binary-elements intercalated graphite superconductors were realized such as RbHgC_8_ (*T*_c_ = 1.44 K)^9^, KTl_1.5_C_4_ (*T*_c_ = 2.7 K)^10^, KTl_1.5_C_8_ (*T*_c_ = 2.5 K)^10^, CsBi_0.55_C_5_ (*T*_c_ = 4.05 K)^11^, Li_3_Ca_2_C_6_ (*T*_c_ = 11.15 K)^12^. Recently, our group succeeded in synthesis of Ca_x_K_1−x_C_8_ (6.5 – 11.5 K) for 0.33 ≤ x ≤ 1^13^. Thus, binary-element intercalation has provided a family of superconductive graphites.

A positive pressure dependence of *T*_c_^onset^ was observed in CaC_6_, and the maximum *T*_c_^onset^ reached 15.1 K at 7.5 GPa^3^. At higher pressure, the *T*_c_^onset^ suddenly dropped. Such a pressure dependence was also observed for other metal-intercalated graphite superconductors. The maximum *T*_c_^onset^ values were 7.1 K at 1.8 GPa for YbC_6_^14^, 2 K at 1 GPa for SrC_6_^6^, and 1.7 K at 1.5 GPa for KC_8_^15^. Such a pressure dependence is characteristic of graphite superconductors. The increase in *T*_c_^onset^ for CaC_6_ was assigned to the softening of the in-plane Ca-Ca phonon and the hardening of the Ca-C phonon^3, 16^. Moreover, the rapid decrease in *T*_c_ is attributed to the order-disorder transition relating to a large softening of the lattice under pressure^17^. Similar behavior under pressure was also observed for binary-elements intercalated graphite Ca_0.6_K_0.4_C_8_, showing a maximum *T*_c_ of 11.6 K at 3.3 GPa^13^.

The mechanism of superconductivity has been extensively discussed based on the theoretical calculation^18, 19^. Calandra and Mauri^18^ suggested clearly that the supecondsuctivity in CaC_6_ is due to an electron-phonon mechanism, and carriers are mostly electrons in the Ca Fermi surface which couples with in-plane Ca-Ca phonon and out-of plane C-C phonons. They suggested the importance of Ca Fermi surface (not π^*^ band of graphite) for the superconductivity. On the other hand, Yang *et al*. experimetally showed the opening of a superconducting gap in the π^*^ band of graphite^19^, suggesting that the superconductivity cannot be assigned to only interlayer band but interaction of π* and interlayer bands. Thus, the mechanism is still under debate. Furthermore, the superconductivity of metal-doped graphene has recently been pursued from theretical and experimental points of view^20–22^. The study on metal-doped graphene may lead to the elucidation of superconductivity in metal-intercalated graphite, since graphene is a thin limit of graphite.

The X-ray diffraction (XRD) patterns of Ca_x_K_1−x_C_y_ (x ≠ 1) suggested a KC_8_-type structure^13^ (face-centered orthorhombic, space group No. 70, *Fddd*)^23^, rather than a CaC_6_-type structure (rhombohedral, space group No. 166, *R* $\bar{3}$m)^2^. The former (KC_8_ structure) shows ‘AαAβAγAδ’, where ‘A’ refers to the graphene sheet, and α, β, γ, and δ refer to the four sites occupied by the metal atoms. On the other hand, the latter (CaC_6_-structure) shows ‘AαAβAγ’ in which metal occupies three different sites. The most interesting point is that in Ca_x_K_1−x_C_y_ the *T*_c_ is much higher than that of KC_8_ despite the KC_8_-type structure.

In this study, we discovered a new binary-elements intercalated graphite superconductor through the intercalation of Ca and Sr. The *T*_c_ values of Ca_x_Sr_1−x_C_y_ with x = 0.8 or 0.9 were ~3 K in the metal-intercalation to highly-oriented pyrolytic graphite (HOPG). Energy dispersive X-ray (EDX) spectroscopy showed the chemical composition of the prepared Ca_x_Sr_1−x_C_y_. The XRD pattern of Ca_x_Sr_1−x_C_y_ showed that the crystal structure is SrC_6_-type (hexagonal, space group No. 194, *P*6_3_/*mmc*)^24^. Therefore, the Ca_x_Sr_1−x_C_y_ sample was finally expressed ‘Ca_x_Sr_1−x_C_6_’. The pressure dependence of the XRD pattern of Ca_x_Sr_1−x_C_6_ showed monotonic shrinkage of the lattice up to 20 GPa. The pressure dependence of *T*_c_ for Ca_x_Sr_1−x_C_6_ showed a positive pressure dependence at 0–8.3 GPa, and a sudden drop in *T*_c_ was observed with applied pressure above 8.3 GPa. The magnetic characteristics of the *R* – *T* plot for Ca_x_Sr_1−x_C_6_ were also studied at 0.80, 4.3 and 8.5 GPa.

## Results

### Preparation and characterization of superconducting Ca_x_Sr_1−x_C_y_ sample through metal doping of HOPG

**Figure 1:**
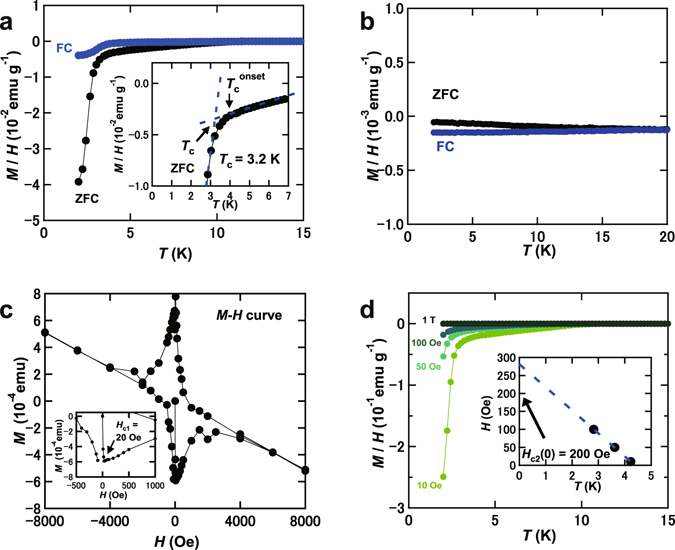
(**a**) *M*/*H* – *T* plots in ZFC and FC modes for Ca_0.8_Sr_0.2_C_y_. (**b**) *M*/*H* – *T* plot in ZFC and FC modes for SrC_6_. (**c**) *M* – *H* plot at 2 K for Ca_0.8_Sr_0.2_C_y_. (**d**) *M*/*H* - *T* plots at different *H’*s for Ca_0.8_Sr_0.2_C_y_, measured in ZFC mode. Inset of (**a**) shows how to determine the *T*_c_. The inset of Fig. 1(c) shows the *M* – *H* plot in the low-*H* range. Inset of (**d**): *H*_c2_ – *T* plot for Ca_0.8_Sr_0.2_C_y_ determined from (**d**). Stoichiometry of Ca_0.8_Sr_0.2_C_y_ refers to the experimental nominal value, and all samples were made by the intercalation of Ca and Sr in HOPG.

**Figure 2:**
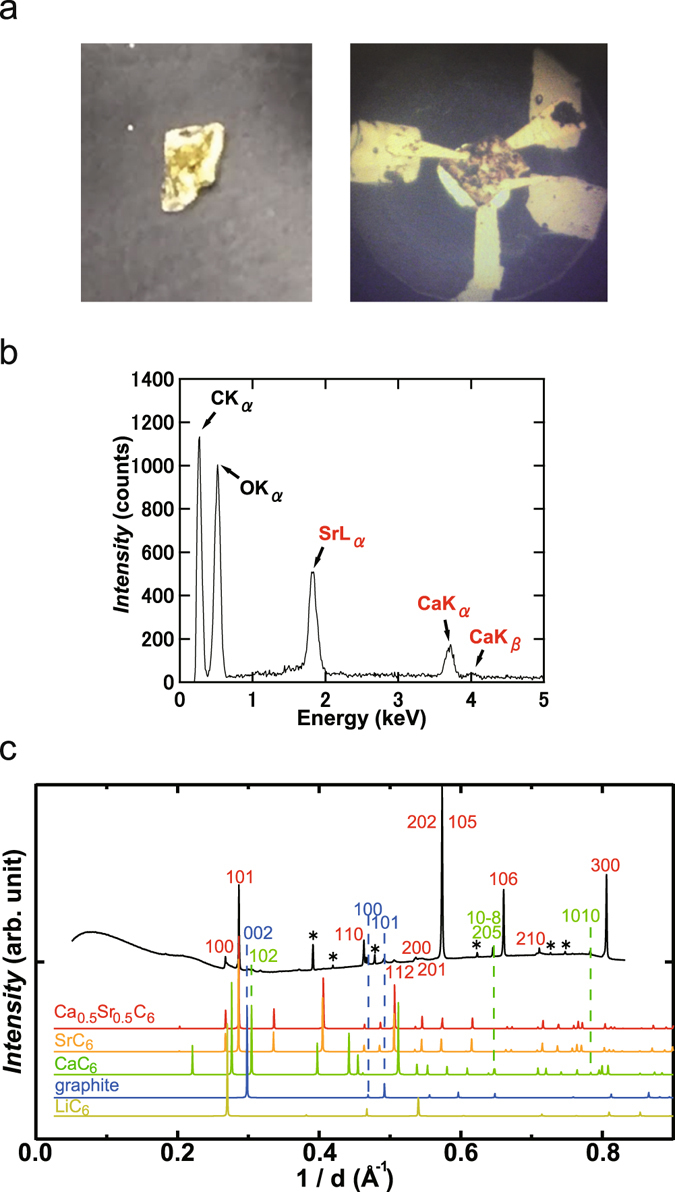
(**a**) Optical image (left) of the Ca_0.8_Sr_0.2_C_y_ sample and microscope image (right) of the sample set in the DAC. (**b**) EDX spectrum of Ca_0.8_Sr_0.2_C_y_. (**c**) XRD pattern of Ca_0.5(2)_Sr_0.5(2)_C_y_. The XRD pattern was measured with synchrotron radiation of λ = 0.68841 Å at room temperature. Patterns shown in (**c**) for Ca_0.5_Sr_0.5_C_6_, SrC_6_, CaC_6_, graphite and LiC_6_ were simulated using the VESTA program, using structural data from refs 2, 24, 25 and 26, respectively.

The temperature (*T*) dependence of magnetic susceptibility, *M*/*H*, (*M*/*H* – *T* plot) measured in zero-field cooling (ZFC) mode in Ca_x_Sr_1−x_C_y_ (x = 0.8) prepared by the intercalation of Sr and Ca to HOPG, which is expressed ‘Ca_0.8_Sr_0.2_C_y_’, is shown in Fig. 1a; *M* and *H* refer to magnetization and applied magnetic field, respectively. The optical image of Ca_0.8_Sr_0.2_C_y_ sample is shown in Fig. 2a, which was bright-gold colour.

A rapid drop in *M*/*H* is observed below ~3.0 K, and *T*_c_ is 3.2 K; how to determine *T*_c_ is shown in the inset of Fig. 1a. The *T*_c_^onset^ is 4.0 K from the *M*/*H* – *T* plot in ZFC mode. The *M*/*H* – *T* plot in field-cooling (FC) mode is shown in Fig. 1a, and the *T*_c_ was also estimated to be 3.2 K. The shielding fraction was estimated to be 100% at 2 K from the *M*/*H* – *T* plot in ZFC mode. Thus, the Ca_0.8_Sr_0.2_C_y_ sample is quite simply a bulk superconductor. On the other hand, we prepared the SrC_6_ sample by the intercalation of Sr in HOPG, which did not show superconductivity down to 2 K, as seen from Fig. 1b. As the *T*_c_^onset^ of SrC_6_ is 1.65 K, the absence of superconductivity is reasonable, suggesting that the Ca_0.8_Sr_0.2_C_y_ sample is not SrC_6_ but Ca/Sr co-doped graphite (Ca_x_Sr_1−x_C_y_).

In this study, we changed nominal x value from 0 to 0.9 in Ca_x_Sr_1−x_C_y_. For Ca_x_Sr_1−x_C_y_ at x ≥ 0.7, the superconductivity was observed. The Ca_x_Sr_1−x_C_y_ sample with nominal x of 0.9, Ca_0.9_Sr_0.1_C_y_, provided both phases of CaC_6_ (*T*_c_ ≈ 11 K) and Ca_x_Sr_1−x_C_y_ (*T*_c_ ≈ 4 K), while that with nominal x of 0.7, Ca_0.7_Sr_0.3_C_y_, showed smaller fraction of superconductivity (*T*_c_ ≈ 2.5 K). For the Ca_x_Sr_1−x_C_y_ sample at nominal x of 0.9, we measured the EDX spectra at eight different positions, which showed three different stoichiometry, Ca_0.98(1)_Sr_0.02(1)_C_y_ (four points), Ca_0.58(6)_Sr_0.42(6)_C_y_ (three points) and Ca_0.35_Sr_0.65_C_y_ (only one point), consistent with two superconducting phases (*T*_c_ ≈ 11 K and *T*_c_ ≈ 4 K) as described above; the Ca_0.35_Sr_0.65_C_y_ is probably lower *T*_c_ than 4 K. Furthermore, for the Ca_x_Sr_1−x_C_y_ sample at nominal x of 0.7, the EDX spectra were measured at five different positions, showing a single phase, Ca_0.2(1)_Sr_0.8(1)_C_y_. This result is consistent with the observation of a single phase exhibiting a small superconducting volume fraction (*T*_c_ ≈ 2.5 K). Thus, owing to the observation of a very large shielding fraction (*T*_c_ = 3.2 K) as shown in Fig. 1a, we investigated the Ca_x_Sr_1−x_C_y_ sample prepared with nominal value of x = 0.8 throughout this study. Finally, we may stress the validity of stoichiometry determined from EDX, based on the consistency between the EDX results and magnetic properties of the Ca_x_Sr_1−x_C_y_ samples.

The *M* – *H* plot of Ca_0.8_Sr_0.2_C_y_ at 2 K is shown in Fig. 1c, which shows typical superconducting *M* – *H* behaviour. The lower critical filed, *H*_c1_, was determined to be 20 Oe (see inset of Fig. 1c). This *H*_c1_ is much smaller than 500 Oe (at 6 K) of CaC_6_^2^. The *M*/*H* – *T* plots at different *H’*s are shown in Fig. 1d. The *H*_c2_ – *T* plot obtained from *M*/*H* – *T* plots (Fig. 1d) is shown in the inset of Fig. 1d, and the *H*_c2_ at 0 K, *H*_c2_(0), was determined to be 200 Oe from the *H*_c2_ – *T* plot using the Werthamer-Helfand-Hochenberg (WHH) formula, *H*_c2_(0) = −0.693*T*_c_(d*H*_c2_/d*T*)_*T*=*T*c_, indicating that the London penetration depth (λ) and Ginzburg Landau coherent length (ξ_GL_) are 215 and 130 nm, respectively. The *H*_c2_ value is much smaller than 7000 Oe of CaC_6_^2^. Here, it should be noted that the *H*_c2_ predicted from the *M* – *H* plot at 2 K (Fig. 1c) seems to be higher than 4000 Oe. This is probably due to the contribution from a CaC_6_ phase, because this sample contains a trace of CaC_6_, as seen from Fig. 1a. This scenario would be reasonable because the *H*_c2_(0) of CaC_6_ is 7000 Oe^2^.

The EDX of Ca_0.8_Sr_0.2_C_y_ is presented in Fig. 2b, and shows peaks due to Sr, Ca, O and C atoms. The presence of O atoms must be due to the oxidation of Ca_0.8_Sr_0.2_C_y_ because the sample used for the EDX spectrum was once treated under atmospheric conditions before the EDX measurement, *i.e*., the contamination of O originates from an extrinsic factor. Therefore, the EDX spectrum suggests that the chemical composition of the Ca_0.8_Sr_0.2_C_y_ sample can be expressed ‘Ca_x_Sr_1−x_C_y_’; the contamination of Li could not be confirmed by the EDX spectrum because the energy of the Li Kα peak is too low. The stoichiometry of Ca_0.8_Sr_0.2_C_y_ was estimated to be Ca_0.5(2)_Sr_0.5(2)_C_y_ from the area intensity of the peaks in the EDX spectrum. Here we can point out that since each peak is substantially resolved in the EDX spectrum (Fig. 2b), the area intensity is obtained with high accuracy. The estimated standard deviation (e.s.d.) of the chemical composition shown above was somewhat large when a large grain of Ca_0.8_Sr_0.2_C_y_ was used for the EDX measurement, indicating that the sample was slightly inhomogeneous. From here, we use the chemical formula, Ca_0.5(2)_Sr_0.5(2)_C_y_, for the Ca_0.8_Sr_0.2_C_y_ sample.

### Structure of superconducting Ca_0.5_Sr_0.5_C_y_

The XRD pattern of Ca_0.5(2)_Sr_0.5(2)_C_y_ at around 0 GPa is shown in Fig. 2c, indicating that the main peaks can be assigned to the SrC_6_-type structure, which is *P*6_3_/*mmc* (No. 194)^24^. Simulation spectra (powder pattern) of LiC_6_, CaC_6_, SrC_6_ and graphite are also shown in Fig. 2c; the simulation was made using the crystal structures of LiC_6_^25^, CaC_6_^2^ SrC_6_^24^, and graphite^26^. Furthermore, as seen from Fig. 2c, the relative intensity of the peaks observed is quite similar to that of SrC_6_. Notably, the XRD pattern was measured with synchrotron radiation (wavelength λ = 0.68841 Å), in which the sample is introduced into a diamond anvil cell (DAC). The pressure was determined to be 0 GPa from the fluorescence of ruby, but the exact pressure may be 0–0.2 GPa.

The lattice constant, *a*, was determined to be 4.32 Å from the *100* and *110* peaks, while the lattice constant, *c*, was determined to be 9.82 Å from the *112* peak using the above *a* value. Furthermore, the values of *a* and *c* were evaluated using iterative approximation. In the iterative approximation, firstly we roughly estimated the *a* value from 100 and 110 peaks. Secondly, the *c* value was estimated from all peaks and the *a* value determined roughly in the first process. Finally the *a* value was estimated from the all peaks and the *c* determined in the second process. The *a* and *c* were 4.31(1) and 9.85(8) Å, respectively. The Le Bail fitting was also tried for determination of *a* and *c*. The *a* and *c* determined by Le Bail fitting were 4.3077(3) and 9.883(1) Å, respectively. Actually, because of the impurity peaks, the Le Bail fitting was difficult. Therefore, these values are for reference. The *a* and *c* values determined by all ways are consistent each other, implying that the *a* and *c* determined were reliable.

It is easy to assume that only 00 l reflections will be measured, if the *ab*-plane of metal-intercalated HOPG sample is completely aligned to the sample holder. However, all reflections are observed as seen from indices of XRD pattern shown in Fig. 2c, indicating that the metal-intercalated HOPG sample is not completely aligned to the sample holder. Therefore, the XRD pattern observed is powder-like with preferred orientation. Consequently, we could successfully obtained both values of *a* and *c*. As seen from Fig. 2c, the XRD pattern of Ca_0.5_Sr_0.5_C_y_ was simulated using *a* = 4.31(1) and *c* = 9.85(8) Å (iterative approximation) and assuming that the 50% of Ca and 50% of Sr randomly occupy the 2*c* site in the space group (No. 194, *P*6_3_/*mmc*) of SrC_6_-type crystal. As seen from the comparison between the experimenmtal XRD pattern and the simulated pattern of Ca_0.5_Sr_0.5_C_6_ (Fig. 2c), most of peaks in the experimental XRD pattern for Ca_0.5(2)_Sr_0.5(2)_C_y_ sample were assigned to those of Ca_0.5_Sr_0.5_C_6_ simulated with SrC_6_ structure. Thus, the indices for most of peaks were provided at SrC_6_ structure, but some peaks were assigned to those of CaC_6_ and graphite. Moreover, some of peaks were not assigned. The difference in relative intensities was found between the experimental XRD pattern and the simulated one of Ca_0.5_Sr_0.5_C_6_, but the conclusion that the sample takes SrC_6_ structure is supported. Furthermore, it should be noticed that to completely reproduce the relative intensities observed in the experimental XRD pattern is difficult, because it shows a powder-like pattern affected by strong preferred orientation, as described above.

The *a* and *c* values are almost the same as those (*a* = 4.316 Å and *c* = 9.88 Å) of SrC_6_^24^, and the simulated pattern of Ca_0.5_Sr_0.5_C_y_ at SrC_6_ structure is consistent with the experimental XRD pattern. As a results, all XRD results support that the stoichiometry of Ca_0.5(2)_Sr_0.5(2)_C_y_ can be expressed ‘Ca_0.5(2)_Sr_0.5(2)_C_6_’. The fact that the Ca/Sr binary-elements intercalated graphite takes the SrC_6_ structure may be reasonable because the ionic radius of Sr^2+^ (1.18 Å for six coordination) is larger than that of Ca^2+^ (1.0 Å for six coordination). Namely, the Ca atoms may be intercalated into the crystal lattice of graphite separated by Sr atoms because of the larger ionic radius of Sr^2+^. In this crystal, the metal atoms occupy two different sites of *α* and *β*, and the graphite layer shows AAA stacking. The stacking form, AαAβAα, of SrC_6_ is different from that, AαAβAγAα, of CaC_6_. The distance, *d*_AA_, between graphenes in SrC_6_ is 4.94 Å (*d*_AA_ = *c*/2), which is larger than the *d*_AA_ = 4.524 Å (*d*_AA_ = *c*/3) in CaC_6_, indicating that the Ca intercalation into SrC_6_ (or Ca_x_Sr_1−x_C_y_) may not affect the lattice constant *c*. In fact, the *c* value of Ca_0.5(2)_Sr_0.5(2)_C_6_ is almost the same as that of SrC_6_, as described above. Thus, despite the SrC_6_ structure, we could obtain the 3 K superconducting phase in Ca_0.5(2)_Sr_0.5(2)_C_6._

Finally, we must comment upon the peaks that cannot be assingned to Ca_0.5_Sr_0.5_C_6_. Some of peaks were assigned to CaC_6_ and pure graphite, as seen from Fig. 2c, indicating the presence of small amount of pure graphite in the sample. This may be the origin of the significant diamagnetic background observed in *M* – *H* plot (Fig. 1c). As described previously, the presence of CaC_6_ in the Ca_0.5(2)_Sr_0.5(2)_C_6_ sample was suggested from the *M*/*H* – *T* plot at ZFC mode shown in Fig. 1a. Here, it should be noticed that the *M*/*H* – *T* at FC mode (Fig. 1a) did not show any trace of CaC_6_. This may imply that the CaC_6_ phase is not bulky but surface (thin layer). As seen from Fig. 2c, some of weak peaks in the XRD pattern were assigned to CaC_6_, indicating the presence of CaC_6_, which is consistent with the observation of a trace of CaC_6_-superconductivity.

### Pressure dependence of superconductivity and structure in Ca_0.5_Sr_0.5_C_6_

**Figure 3:**
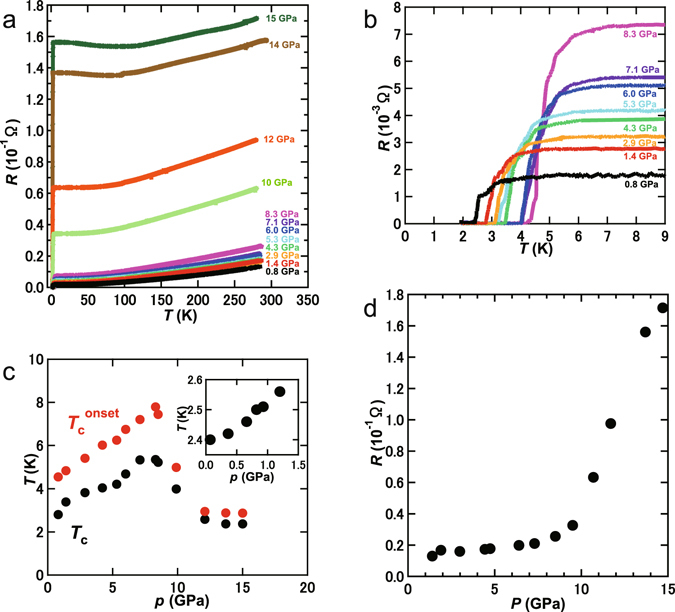
*R* – *T* plots for Ca_0.5(2)_Sr_0.5(2)_C_6_ at different pressures in *T* range of (**a**) 2–300 K and (**b**) 2–9 K. (c) *T*_c_ – *p* and (**d**) *R* – *p* plots for Ca_0.5(2)_Sr_0.5(2)_C_6_; *R* in (**d**) means the *R* value at 280 K. Inset of (**c**): *T*_c_ – *p* plot for the Ca_0.9_Sr_0.1_C_y_ sample below 1.5 GPa, which was determined from *M*/*H* – *T* plots at different pressures; this sample’s stoichiometry is shown in the text.

Microscope image of Ca_0.5(2)_Sr_0.5(2)_C_6_ sample and four electrodes set in DAC is shown in Fig. 2a, in which four electrodes are contacted to the sample. The sample shows bright-gold color. Figure 3a and b show the temperature dependence of resistance (*R* – *T* plots) of Ca_0.5(2)_Sr_0.5(2)_C_6_ at different pressures. The former shows the *R* – *T* plots at 2–300 K, and the latter shows the expanded plots (2–9 K). The pressure dependence of *T*_c_ in Ca_0.5(2)_Sr_0.5(2)_C_6_ is shown in Fig. 3c; the *T*_c_ was determined from the cross point of the *R* – *T* plot at normal state and that exhibiting the drop, in the same manner as the inset of Fig. 1a. The *T*_c_ increased with increasing pressure up to 8.3 GPa, then suddenly decreased. This behaviour is similar to that of CaC_6_^3^ and Ca_0.6_K_0.4_C_8_^13^. Such a positive pressure dependence of *T*_c_ in Ca_0.5(2)_Sr_0.5(2)_C_6_ may also be due to the softening of in-plane Ca(Sr)-Ca(Sr) phonons, as suggested in CaC_6_^3^. The highest *T*_c_ was 5.4 K at 8.3 GPa. The values of d*T*_c_/d*p* and d*T*_c_^onset^ /d*p* were determined to be 0.34(4) K GPa^−1^ and 0.42(1) K GPa^−1^, respectively, from the *T*_c_ – *p* and *T*_c_^onset^ – *p* plots at 0–8.3 GPa, consistent with those of SrC_6_ and CaC_6_ (d*T*_c_/d*p* (SrC_6_) = 0.35 K GPa^−1^ and d*T*_c_^onset^ /d*p* (CaC_6_) = 0.39 K GPa^−1^)^6, 27^. The sudden drop of *T*_c_ is found in CaC_6_ and Ca_0.6_K_0.4_C_8_ which was assigned to the order-disorder transition originating from random off-center displacement of Ca(K) atoms in the *ab*-plane with accompanying lattice-softening^3, 17^. Therefore, the *T*_c_ drop in the pressure range above 8.3 GPa for Ca_0.5(2)_Sr_0.5(2)_C_6_ may be assigned to the above order-disorder transition.

**Figure 4:**
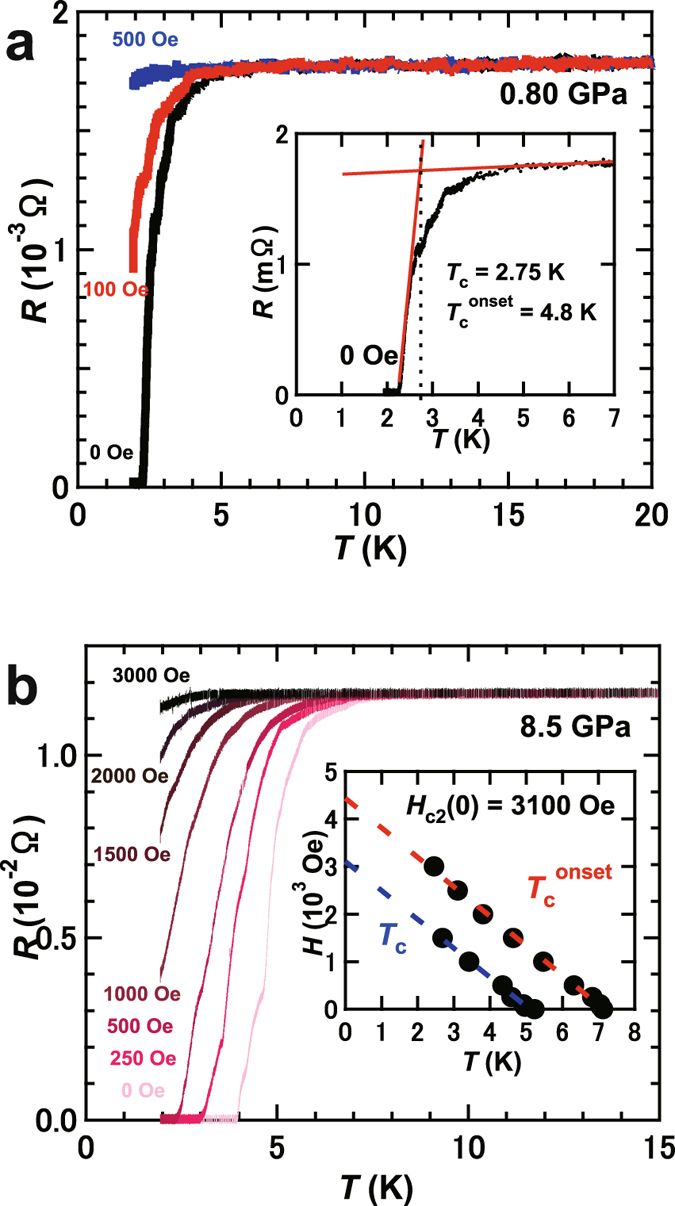
*R* – *T* plots of Ca_0.5(2)_Sr_0.5(2)_C_6_ at different *H’*s under pressure of (**a**) 0.80 GPa and (**b**) 8.5 GPa. Inset of (**a**) shows how to determine *T*_c_, and inset of (**b**) shows plots of *H* – *T*_c_^onset^ and *H* – *T*_c_ for Ca_0.5(2)_Sr_0.5(2)_C_6_. The *H* – *T*_c_^onset^ refers to *H*_c2_ – *T* plot.

The *R* – *T* plots at *H’*s of 0 and 500 Oe were measured at 0.80 GPa (Fig. 4a), indicating the suppression of superconductivity at 500 Oe. Furthermore, the *R* – *T* plots at different *H* values were measured at 4.3 and 8.5 GPa. Figure 4b shows the *R* – *T* plots at different *H* values at 8.5 GPa. The *H*_c2_ – *T* plot determined from the graph shown in Fig. 4b is depicted in the inset of Fig. 4b. The *H*_C2_(0) at 8.5 GPa was evaluated to be 3100 Oe from the WHH formula. This value is larger than that, 200 Oe, evaluated from *M*/*H* – *T* plots at 0 GPa (inset of Fig. 1d). Notably, as seen from Fig. 3a, the behavior of the *R* – *T* plot in the normal state was metallic up to 12 GPa, *i.e*., the *R* decreased with decreasing temperature. But at 14 and 15 GPa, the *R* increased slightly with decreasing temperature below 90 K, suggestive of a change in electric transport in the normal state at around 14 GPa (Fig. 3a). The *M*/*H* – *T* plots at different pressures (0–1.3 GPa) for Ca_0.9_Sr_0.1_C_y_ are shown in Fig. 1S of Supplementary Information, showing the positive pressure dependence. This sample contained three diffrenet phases, Ca_0.98(1)_Sr_0.02(1)_C_y_, Ca_0.58(6)_Sr_0.42(6)_C_y_ and Ca_0.35_Sr_0.65_C_y_, as shown previously, but the stoichiometry exhibiting the *T*_c_’s determined from the *M*/*H* – *T* plots (Figure S1) would be Ca_0.58(6)_Sr_0.42(6)_C_y_ which is almost the same as Ca_0.5(2)_Sr_0.5(2)_C_y_. The *T*_c_ – *p* plot obtained from *M*/*H* – *T* at 0–1.3 GPa is shown in the inset of Fig. 3c. Figure 3d shows the pressure dependence of *R* at 280 K for Ca_0.5(2)_Sr_0.5(2)_C_6._ The *R* rapidly increases above 10 GPa, which may be correlated with the change in electric transport above 12 GPa shown in Fig. 3a.

**Figure 5:**
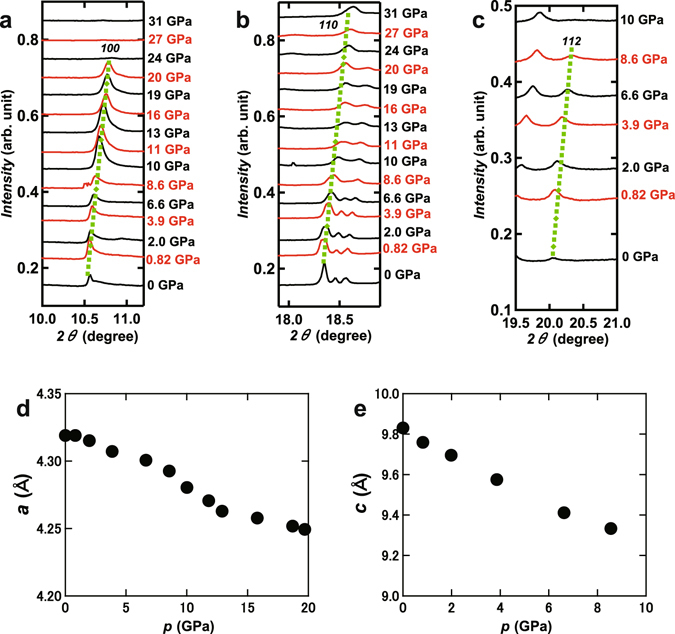
Pressure dependence of peaks ascribable to (**a**) *100*, (**b**) *110* and (**c**) *112* for Ca_0.5(2)_Sr_0.5(2)_C_6_. (**d**) Pressure dependence of lattice constants, (**d**) *a* and (**e**) *c*, for Ca_0.5(2)_Sr_0.5(2)_C_6_.

Figure 5a–c show the pressure dependence of three representative peaks of Ca_0.5(2)_Sr_0.5(2)_C_6_ in the XRD pattern. These peaks shifted to higher 2*θ* with an increase in pressure, indicating shrinkage of the unit cell. The *100* peak was observed up to 20 GPa, but suddenly disappeared above 20 GPa, while the *110* peak was clearly observed across the entire range of applied pressure (0–31 GPa). Moreover, the *112* peak quickly disappeared above 8.6 GPa. In Ca_0.6_K_0.4_C_8_, the *004* peak completely disappeared at 16 GPa^13^, which was assigned to the structural change from the KC_8_ structure to a non-graphite type structure. The disappearance of the *112* peak at 10 GPa would be attributed to the vanishing of the long-range order of graphite, such as the graphite – non-graphite transition found at around 16 GPa in Ca_0.6_K_0.4_C_8_^13^. Furthermore, the change of electric transport (Fig. 3a) and the rapid increase in *R* (Fig. 3d) may be explained by considering a structural transition at around 10 GPa.

**Figure 6:**
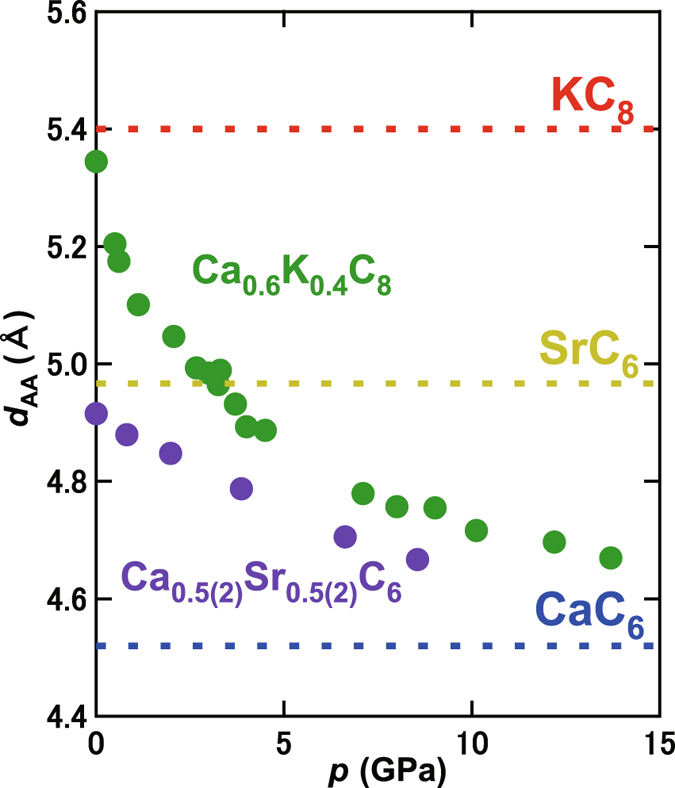
Pressure dependence of *d*_AA_ for Ca_0.6_K_0.4_C_8_ and Ca_0.5(2)_Sr_0.5(2)_C_6_. Dashed lines drawn in red, yellow and blue refer to the *d*_AA_ values of KC_8_, SrC_6_ and CaC_6_, respectively.

The pressure dependence of lattice constants *a* and *c* is plotted in Fig. 5d and e; the *a* was determined up to 20 GPa, while *c* determined up to 8.6 GPa because of the rapid disappearance of the *112* peak around 10 GPa. Both plots show a monotonic shrinkage of the unit cell with increasing pressure. The pressure dependence of *d*_AA_ in Ca_0.6_K_0.4_C_8_ and Ca_0.5(2)_Sr_0.5(2)_C_6_ is shown in Fig. 6; that of Ca_0.6_K_0.4_C_8_ is taken from ref. 13. The behaviour of *d*_AA_ – *p* is similar in both. Namely, the *d*_AA_ approaches the *d*_AA_ (=4.524 Å) of CaC_6_ with increasing pressure, and any Bragg peak disappears when reaching that *d*_AA_ (above 13.7 GPa for Ca_0.6_K_0.4_C_8_ and above 8.6 GPa for Ca_0.5(2)_Sr_0.5(2)_C_6_). To sum up, any structural transition may take place when the *d*_AA_ reaches the threshold value of *d*_AA_.

## Discussion

In this paper, the most important issue is that a new class of superconducting binary-elements intercalated graphite was prepared by the intercalation of Sr and Ca. These are alkali-earth elements, and their ionic radii differ slightly (Sr^2+^: 1.18 Å for six coordination and Ca^2+^: 1.0 Å for six coordination). The ionic radii of some elements which can be intercalated to graphite are shown in Table 1, in which they were taken form ref. 28. On the other hand, the crystal structure is different between CaC_6_ and SrC_6_, in which the former takes the rhombohedral structure (space group No. 166, *R* $\bar{3}$m)^2^, while the latter takes the hexagonal structure (space group No. 194, *P*6_3_/*mmc*)^24^. In both crystals, the graphene sheets stack in AAA form, but location of Ca or Sr is different; AαAβAγA for CaC_6_, and AαAβA for SrC_6_. Previously, we successfully made the superconducting Ca_x_K_1−x_C_y_ materials which consist of alkali and alkali earth elements. The ionic radii of Ca and K are 1.0 Å (for six coordination) and 1.38 Å (for six coordination), respectively, which are quite different. The crystal of KC_8_ takes face-centered orthorhombic structure (space group No. 70, *Fddd*), in which the stacking form is AαAβAγAδA, different from that of CaC_6_. Regardless of such a large difference between CaC_6_ and KC_8_, Ca_x_K_1−x_C_y_ was successfully formed.

**Table 1:** Ionic radius of elements (from ref. 28).

Element	Coordination number	Ionic radius (Å)
Li	6	0.76
K	6	1.38
Cs	6	1.67
Ca	6	1.0
Sr	6	1.18
Yb	6	1.02

**Figure 7:**
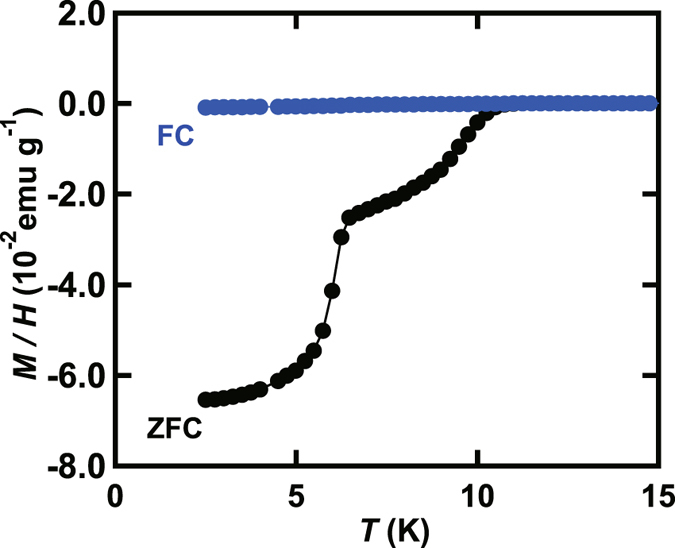
*M*/*H* – *T* plots in ZFC and FC modes for Ca_0.8_Yb_0.2_C_y_. Presence of two phases of CaC_6_ and YbC_6_ is indicated.

On the other hand, we tried to fabricate Ca_x_Yb_1−x_C_y_, but the *M*/*H* – *T* plot showed a complete phase separation of CaC_6_ (*T*_c_ = 11.5 K) and YbC_6_ (*T*_c_ = 6.7 K), as seen from Fig. 7. The crystal structure of YbC_6_ is the same as that of SrC_6_. The ionic radius of Yb is 1.02 Å for six coordination, which is the same as that of Ca. Nevertheless, the Ca_x_Yb_1−x_C_y_ could not be realized thus far. The liquid alloy method has been used for the preparation of binary-elements intercalated graphites, and the YbC_6_ and CaC_6_ phases were separately generated in the preparation of Ca_x_Yb_1−x_C_y_, suggesting both elements are melted. Therefore, we can rule out the possibility of no melting of either element.

Here, we focus on the fact that the element with the larger ionic radius dominates the crystal structure, *i.e*., the SrC_6_ structure in Ca_x_Sr_1−x_C_y_ and the KC_8_ structure in Ca_x_K_1−x_C_y_. Furthermore, the *d*_AA_ in binary-elements intercalated graphite is the same as that of a crystal lattice consisting solely of an element with larger ionic radius; the *d*_AA_ (=c /2 = 4.91 Å (*c* = 9.81 Å) or 4.925 Å (*c* = 9.85(8) Å) of Ca_x_Sr_1−x_C_y_ is the same as that (=*c*/2 = 4.95 Å) of SrC_6_, and the *d*_AA_ = (c /4 = 5.40 Å) of Ca_x_K_1−x_C_8_ is the same as that (=c/4 = 5.35 Å)^23^ of KC_8_, as seen from Fig. 2 of ref. 23. These facts may point to a scenario in which the crystal lattice formed by the element with larger ionic radius is subsequently doped with the other element with smaller ionic radius. Based on this scenario, we can propose suitable combinations for the superconducting binary-elements or ternary-elements intercalated graphites, *i.e*., the binary-elements graphites must be realized using Cs and Ca, or Cs and Yb, because of the larger difference in ionic radii (Cs^+^: 1.67 Å for six coordination), and for the ternary-elements superconductors the combination of Ca (or Yb), Sr (or K) and Cs are probably suitable. The crystal structure of the binary- and ternary-elements intercalated graphites suggested above would be the CsC_8_-type structure, because the CsC_8_ phase is formed with the CsC_8_ structure^29^.

## Methods

### Sample preparation and characterization

The Ca_x_Sr_1−x_C_y_ samples were prepared using the liquid-alloy method. Ca and Sr metals were mixed in appropriate molar ratios and placed in an iron vessel with Li. The molar ratio of Li was the same as the sum of Ca and Sr. The vessel was then heated to 350 °C, at which temperature the Ca/Sr/Li alloy was completely melted. The HOPG was immersed in the molten Ca/Sr/Li alloy for approximately one week. The whole preparation was performed in an Ar-filled glove box (O_2_ and H_2_O concentrations were maintained below 0.1 ppm). The *M*/*H – T* curves of the Ca_x_Sr_1−x_C_y_ samples were measured with a SQUID magnetometer (Quantum Design, MPMS2). All XRD patterns at 0–31 GPa were measured at 295 K using synchrotron radiation (λ = 0.68841 Å) at BL12B2 of SPring-8. The simulated XRD patterns for LiC_6_, CaC_6_ and SrC_6_ made using the VESTA program^30^ were employed for the analyses of XRD patterns.

The diamond anvil cell was used for the measurements, and the Ca_0.5(2)_Sr_0.5(2)_C_6_ sample was placed in the diamond anvil cell without any exposure of the sample to air, as is described below. A 300-μm-thick stainless steel gasket with a 160-μm diameter hole was placed on a diamond with a 400-μm culet, and the sample was introduced into the hole. The sample was covered with daphne oil (Idemitsu Co., Ltd., Daphne 7373) as the pressure medium. Finally the sample was pressed by another diamond. The pressure was monitored by the fluorescence peak of a piece of ruby set in the DAC. The pressure dependence of the *M*/*H* – *T* plot for Ca_x_Sr_1−x_C_y_ was measured using the above SQUID equipment in which the sample was placed in a piston-cylinder cell; the pressure medium was the same daphne oil as above. Meanwhile, the pressure dependence of the *R* – *T* plots was measured in four-terminal measurement mode; the used sample was identified to be Ca_0.5(2)_Sr_0.5(2)_C_6_. The sample was placed in a diamond anvil cell (DAC); the pressure medium was NaCl. Details of sample-setting for the *M*/*H* – *T* and *R* – *T* measurements at high pressure are described elsewhere^13^. The *R* was recorded using an AC resistance-bridge (Lakeshore, 370-type Resistance Bridge), limiting the applied current to less than 100 µA. The sample was cooled using liquid He, and the temperature was controlled with a temperature controller (Oxford, ITC503 Temperature Controller).

## Electronic supplementary material

Supplementary Information
