# Supplementary material for: Preparation and characterization of a new graphite superconductor: Ca0.5Sr0.5C6
Source: Sci Rep. 2017 Aug 7;7:7436. doi: 10.1038/s41598-017-07763-y (PMC5547157; doi:10.1038/s41598-017-07763-y)
Supplement: Supplementary file 1 — Supplementary Information [file 41598_2017_7763_MOESM1_ESM.doc]

Supplementary Information

**Preparation and characterization of a new graphite superconductor: Ca_0.5_Sr_0.5_C_6_**

Saki Nishiyama,^1^ Hidenori Fujita,^2^ Masatoshi Hoshi,^2^ Xiao Miao,^1^ Takahiro Terao,^1^ Xiaofan Yang,^1^ Takafumi Miyazaki,^3^ Hidenori Goto,^1^ Tomoko Kagayama,^2^ Katsuya Shimizu,^2^ Hitoshi Yamaoka,^4^ Hirofumi Ishii,^5^ Yen-Fa Liao^5^ and Yoshihiro Kubozono^1*^

^1^Research Institute for Interdisciplinary Science, Okayama University, Okayama 700-8530, Japan

^2^[Center for Science and Technology under Extreme Conditions](http://www.cqst.osaka-u.ac.jp/), Graduate School of Engineering Science, Osaka University, Osaka 560-8531, Japan

^3^Research Laboratory for Surface Science, Okayama University, Okayama 700-8530, Japan

^4^RIKEN SPring-8 Center, RIKEN, 1-1-1 Kouto, Sayo, Hyogo 679-5148, Japan

^5^National Synchrotron Radiation Research Center, Hsinchu 30076, Taiwan


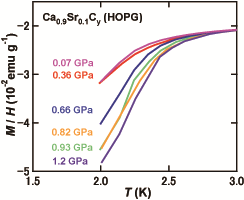


Figure S1. *M* / *H* – *T* plots of Ca_0.9_Sr_0.1_C_y_ (HOPG) at different pressures.
